# Eph Regulates Dorsoventral Asymmetry of the Notochord Plate and Convergent Extension-Mediated Notochord Formation

**DOI:** 10.1371/journal.pone.0013689

**Published:** 2010-10-29

**Authors:** Izumi Oda-Ishii, Yasuo Ishii, Takashi Mikawa

**Affiliations:** Cardiovascular Research Institute, University of California San Francisco, San Francisco, California, United States of America; National University of Singapore, Singapore

## Abstract

**Background:**

The notochord is a signaling center required for the patterning of the vertebrate embryic midline, however, the molecular and cellular mechanisms involved in the formation of this essential embryonic tissue remain unclear. The urochordate *Ciona intestinalis* develops a simple notochord from 40 specific postmitotic mesodermal cells. The precursors intercalate mediolaterally and establish a single array of disk-shaped notochord cells along the midline. However, the role that notochord precursor polarization, particularly along the dorsoventral axis, plays in this morphogenetic process remains poorly understood.

**Methodology/Principal Findings:**

Here we show that the notochord preferentially accumulates an apical cell polarity marker, aPKC, ventrally and a basement membrane marker, laminin, dorsally. This asymmetric accumulation of apicobasal cell polarity markers along the embryonic dorsoventral axis was sustained in notochord precursors during convergence and extension. Further, of several members of the *Eph* gene family implicated in cellular and tissue morphogenesis, only *Ci-Eph4* was predominantly expressed in the notochord throughout cell intercalation. Introduction of a dominant-negative Ci-Eph4 to notochord precursors diminished asymmetric accumulation of apicobasal cell polarity markers, leading to defective intercalation. In contrast, misexpression of a dominant-negative mutant of a planar cell polarity gene *Dishevelled* preserved asymmetric accumulation of aPKC and laminin in notochord precursors, although their intercalation was incomplete.

**Conclusions/Significance:**

Our data support a model in which in ascidian embryos Eph-dependent dorsoventral polarity of notochord precursors plays a crucial role in mediolateral cell intercalation and is required for proper notochord morphogenesis.

## Introduction

Patterning along the midline body axis in vertebrates depends upon signals from a transient embryonic tissue, the notochord [1], [2], [3]. This tissue develops from a precursor population that is specified at the posterior midline and elongates anteroposteriorly along the embryonic midline through complex morphogenetic processes during gastrulation and neurulation [4], [5], [6]. Pioneer studies in frog embryos have revealed that cell intercalation perpendicular to the anteroposterior axis, known as convergence and extension, plays a key role in notochord elongation without volume change [7].

Several molecular components involved in this morphogenetic movement during notochord formation have been identified. These include members of the planar cell polarity gene family and the *Eph/ephrin* gene family [8], [9]. Altered expression of these factors causes defects in convergence and extension without affecting cell differentiation [10], [11], [12], [13], [14], [15]. A dominant negative form of Xenopus Dishevelled, XDshD2, impairs convergent extension and PCP signaling but not canonical Wnt pathway when misexpressed in Xenopus embryos [16], [17]. Introduction of XDshD2 in *Ciona* notochord cells results in abnormal cell intercalations [18]. A truncated form of Eph receptor, which lacks an intracellular protein tyrosine kinase domain, blocks Eph signaling in various organisms [19], [20], [21], [22] and causes morphological defects of the notochord in zebrafish [10]. However, due in part to structural complexity of the notochord of higher vertebrates, which is composed of multiple rows of cells, our understanding as to how the above molecules regulate notochord elongation or which step(s) of the convergence-and-extension mechanism is their target(s) still remains rudimentary. A simpler animal model may facilitate our analysis of cellular and molecular mechanisms that regulate notochord morphogenesis.

Ascidians, which are primitive chordates, establish a notochord that consists of only 40 cells aligned in a single cell diameter column [23]. Ascidian notochord cells originate from a monolayer of 40 postmitotic mesoderm cells, called the notochord plate. As gastrulation proceeds, notochord plate cells undergo horizontal sliding and intercalate with each other mediolaterally, without dorsoventral sliding. This horizontally restricted cell-cell intercalation and a concomitant invagination of the notochord plate generates the single cell array of the mature notochord along the embryonic midline [24], [25], [26]. The relative simplicity of the ascidian notochord, compared to higher vertebrates, therefore allows for detailed study of the mechanisms underlying notochord morphogenesis potentially through analysis of precursor cell horizontal intercalation.

The mediolateral cell intercalation of the notochord plate requires PCP pathway components [27]. It has also been shown that notochord precursor explants differentiate autonomously but require neighboring tissues to undergo cell rearrangement and elongation, suggesting that notochord development involves both cell autonomous and non-autonomous mechanisms [28], [29]. Using embryos of the urochordate *Ciona intestinalis* as a model system, the present study addresses both the cellular and molecular basis that prohibits notochord precursors from dorsoventral sliding and supports their intercalation. Evidence is presented to show that notochord plate cells exhibit asymmetric accumulation of apicobasal polarity markers along the dorsoventral axis, and that this polarity is maintained throughout subsequent cell intercalation process. While the *Ciona* genome contains several members of the *Eph* gene family [30], we show that only *Ci-Eph4* is expressed predominantly in notochord plate cells throughout notochord cell intercalation. We provide evidence that reduced *Ci-Eph4* function results in defective cell intercalation and mislocalization of apicobasal markers. Our study in the primitive chordate *Ciona* provides a framework for understanding evolutionarily conserved and/or diversified molecular and cellular mechanisms critical for midline formation in chordates.

## Results

### Notochord plate cells asymmetrically accumulate apicobasal polarity markers along the dorsoventral axis

**Figure 1:**
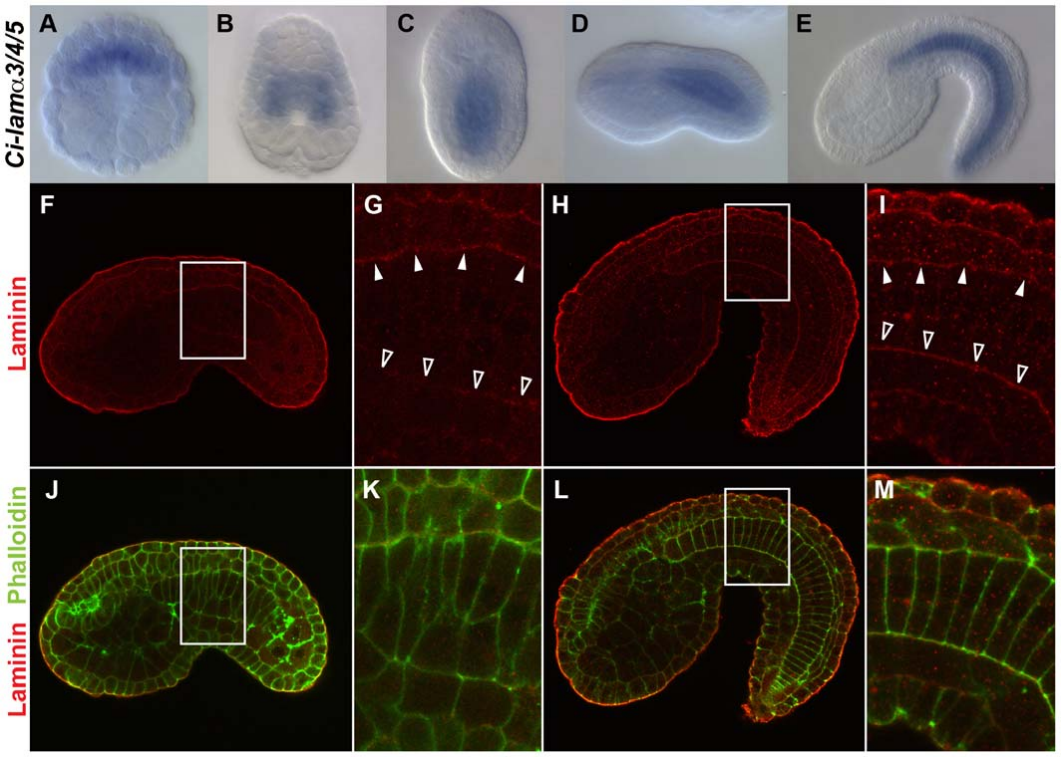
Distribution of laminin in *Ciona intestinalis* embryos. (A–E) Whole-mount *in situ* hybridization of early gastrula (A), late gastrula (B), late neurula (C), early tailbud (D) and middle-late tailbud (E) stages for *Ci-lamα3/4/5 mRNA*. A is vegetal view, B and C are dorsal views, and D and E are lateral views. Anterior is to the top in A–C and to the left in D and E. Note that *Ci-lamα3/4/5* is preferentially expressed in notochord lineage cells. (F–M) Confocal images of embryos stained with the anti-Cs-lamα3/4/5 (red) alone (F–I) and double staining with phalloidin (green, J–M) at early tailbud (F,G,J,K) and middle-late tailbud (H,I,L,M) stages. All images are lateral views and anterior is to the left. G, I, K and M are higher magnification of the boxed area in F, H, J and L, respectively. Note: In all cases, high background red signal outlining embryos was seen perhaps due to an edge effect as *Cs-lamα3/4/5* mRNA is undetectable there. At early taulbud stage, a high signal accumulation or maternal laminin protein is detectable at the dorsal side (white arrowheads in G) but not at the ventral side (open arrowheads in G) of the notochord. When intercalation is completed, Cs-lamα3/4/5 signal expands to the ventral side (open arrowheads in I) of the notochord and asymmetric distribution along the dorsoventral axis is lost (I,M).

The specific directional intercalation of notochord plate cells along the mediolateral axis of ciona intestinalis can potentially be explained by several mechanisms, such as a tight intercellular connection [25] and/or a physical barrier that suppresses notochord cell movement along a dorsoventral direction [31], [32], [33]. Our survey of cell junction components, however, failed to detect any specific tight or adherent junctions highly accumulated in notochord plate cells (data not shown). Indeed, an increased intercellular connection would be potentially destructive for notochord plate cells which undergo active cell intercalation for notochord elongation. Therefore, we examined a potential physical barrier that would restrict notochord plate cell movement to a lateral-to-medial direction. It has recently been shown that transcripts of a basal lamina component laminin, *Cs-lamα3/4/5*, are highly expressed in the notochord of *Ciona savignyi* embryos and that its perichordal accumulation is necessary for normal notochord development [34]. Notochord cells complete their final cell division at the neurula stage and start mediolateral intercalation. This intercalation movement continues until the end of early tailbud stage [35]. To test if the *laminin* gene is expressed in the notochord plate of *Ciona intestinalis* embryos, we examined expression of its *Ciona intestinalis* ortholog *Ci-lamα3/4/5* by whole mount in situ hybridization. *Ci-lamα3/4/5* transcripts were detected in notochord lineage cells prior to (Figure 1A,B) and throughout mediolateral cell intercalation (Figure 1C–E).

To examine distribution of laminin proteins in the *Ciona intestinalis* notochord, embryos at various stages of notochord plate cell intercalation [35] were stained with an antibody raised against Cs-lamα3/4/5 protein. Weak but higher signal than background of Laminin became detectable at the late neurula stage. Importantly, the laminin staining signal was more prominent at the dorsal surface of the notochord when notochord cells undergo active intercalation (Figure 1F,G,J,K). After completion of convergence and extension, laminin was detected over the whole surface of the notochord (Figure 1H,I,L,M) as reported in *Ciona savigni* [34]. Similar localized signal was also detected by immunostaining with an antibody raised against purified mammalian laminin protein from the basement membrane of mouse Englebreth Holm-Swarn sarcoma, though there was higher background signal, consistent with our Western blotting analysis that showed that its lower specificity than Cs-lam*α*3/4/5 antibody (Figure S1). Taken together, the data suggest that the notochord plate during mediolateral intercalation exhibits asymmetric distribution of a basal lamina component, laminin.

**Figure 2:**
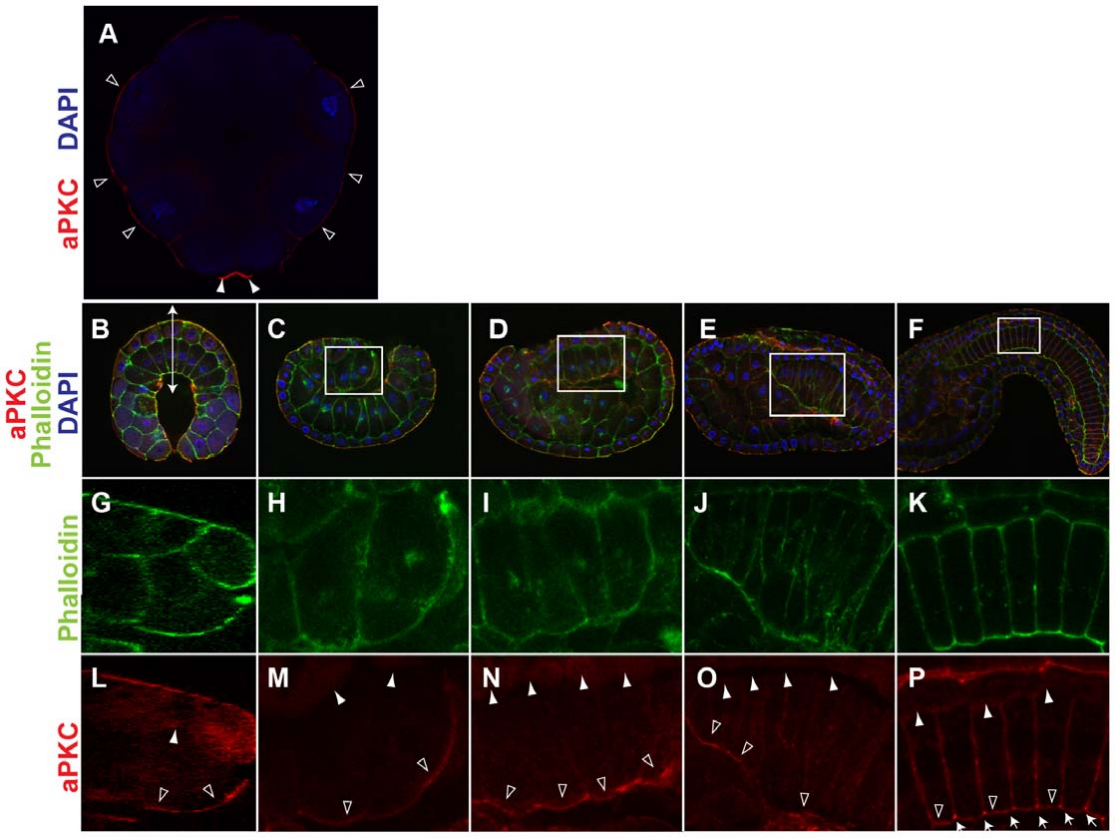
Asymmetric distribution of aPKC along the dorsoventral axis of notochord cells. (A) Confocal section image of a 16-cell stage embryo double-stained with anti-aPKC (red) and DAPI (blue). Note aPKC accumulattion to the outer surface of all blastmeres (open arrowheads) in addition to high levels to the centrosome attracting body (arrowheads). Vegetal view, anterior is to the top. (B–F) Confocal section images of early gastrula (B), late gastrula (C) middle neurula (D), early tailbud (E) middle-late tailbud (F) stage embryos triple-stained for aPKC (red), phalloidin for actin (green) and DAPI for nuclei (blue). B is a vegetal view with anterior to the top, and C–F are lateral views with anterior to the left. (G) Sagittal section image of phalloidin staining of the area indicated by a double-arrow in B. (H–K) Higher magnification phalloidin staining images of the boxed areas in C–F, respectively. (L–P) As in G–H but aPKC staining confocal images. Note that aPKC is preferentially accumulated at the vegetal/ventral side (blank arrowheads in L–P) than the animal/dorsal side (white arrowheads in L–P) of notochord precursors. In middle-late tailbud stage embryos, aPKC is highly localized at the boundary between individual notochord cells (arrows in P).

Asymmetric distribution of laminin lead to the hypothesis that notochord plate may be polarized along the dorsoventral axis, similar to the apicobasal polarity of epithelial cells. To test this possibility, we examined localization of an apical polarity marker, aPKC [36], [37], [38], [39], [40]. In contrast, aPKC showed a clear asymmetric localization in notochord cells along the embryonic dorsoventral axis (Figure 2). At 16-cell stage, weak but higher levels of aPKC signal than the background was detected in the outer or apical surface of all blastomeres (Figure 2A) in addition to the previously reported accumulation to the centrosome attracting body [41]. The outer, apical accumulation of aPKC became more evident in early gastrula embryos (Figure 2B,G,L). Notochord precursors, which are located in the vegetal layer, showed a strong aPKC signal at their outer surface (Figure 2L; open arrowheads). As gastrulation proceeds, notochord precursors invaginate and constitute a part of the archenteric roof. An apical or ventral accumulation of aPKC was detected in these cells (Figure 2C,H,M). This asymmetric accumulation was retained late in gastrulation and during notochord cell intercalation (Figure 2D,I,N) even after the archenteron space between mesoderm and endoderm becomes unrecognizable (Figure 2E,J,O,F,K,P and S2A–C). Once notochord plate cell intercalation was completed, aPKC localization was shifted to the center of the boundary between notochord cells (Figure S2D–I) where an extracellular lumen develops to form a hollow notochord as previously reported [26].

**Figure 3:**
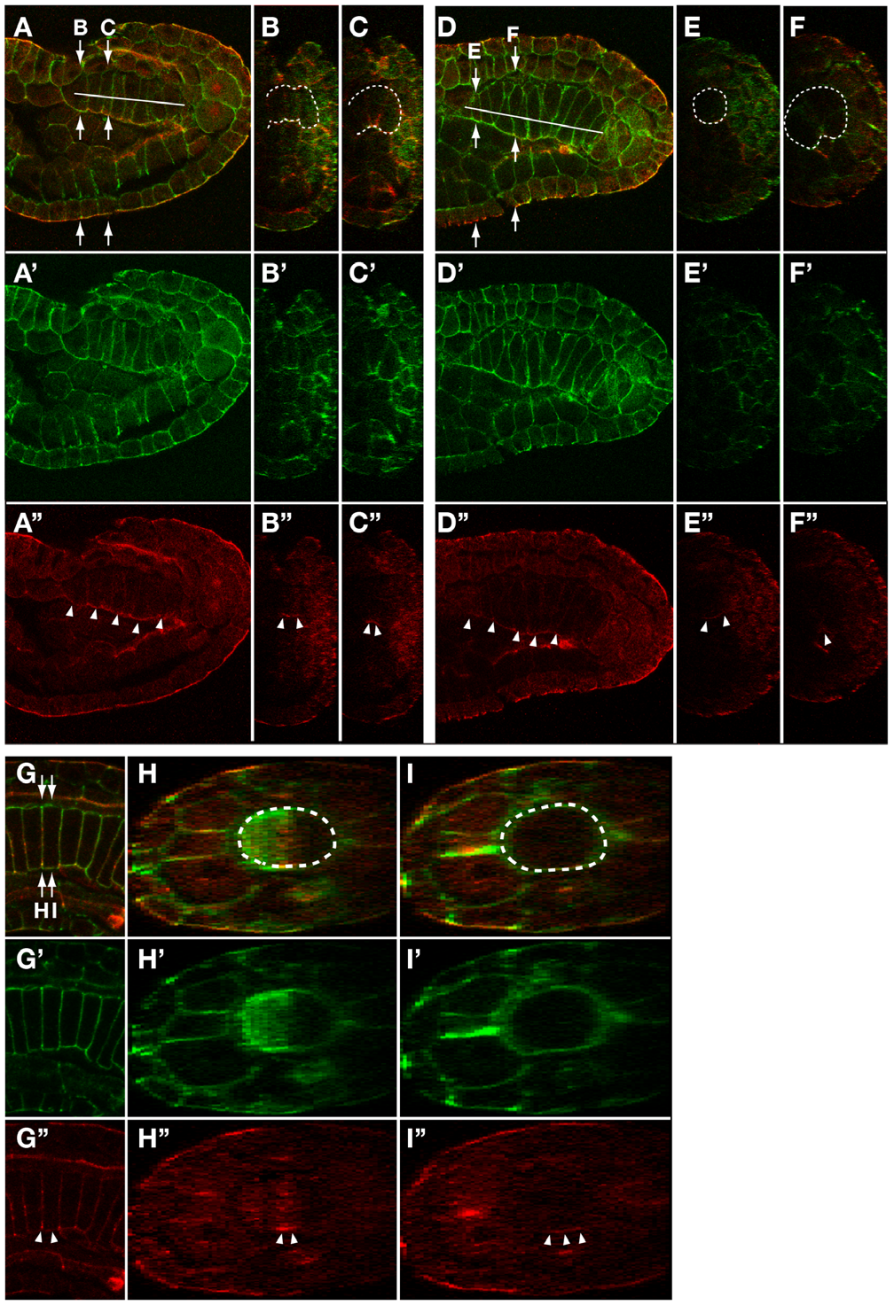
Accumulation of aPKC at the ventral surface of notochord cells is retained during cell intercalation process. (A,D,G) Confocal saggital section images of the notochord of a late neurula (A), early tailbud (D) and middle-late tailbud (G) stage embryos double-stained for aPKC (red) and phalloidin for actin (green). Lateral views with anterior to the left. Reconstructed cross section image at the level indicated by arrows in A, D and G is shown in B/C, E/F and H/I. Position of the notochord in saggital section and cross section images is indicated by a white line in A and D or surrounded by white dotted line in B,C,E,F,H,I. Accumulation of aPKC is indicated by white arrowheads in A’–I’.

To obtain more detailed information about aPKC localization, we captured confocal z-stack images in late-neurula and early-tailbud stage embryos and reconstructed sections perpendicular to the AP axis. Z-stack images were also compiled into movies to survey all z-stack images (Movies S1 and S2). These data confirmed strong aPKC signal localization at the ventral surface of the notochord plate, which narrows as the notochord plate invaginates and faces to the remnant of the archenteron (Figure 3B,C) or the endoderm (Figure 3E,F). The signal was not detected over the lateral surface facing to muscle cells (Figure 3C,F). Comparison between aPKC staining and cell boundaries (as shown by phalloidin staining) in Supplemental movies S1 and S2, demonstrate that most, if not all, notochord cells show strong aPKC signal on their ventral surface. Subsequent to cell intercalation, high accumulation of aPKC signal was still detectable on the ventralmost surface of notochord cells but not on the dorsal and lateral surfaces (FIgure 3G–I). These data show that the notochord plate asymmetrically localize apicobasal cell polarity markers along the embryonic D–V axis during convergence and extension.

### *Ci-Eph4* is expressed in the notochord cell lineage throughout convergence and extension

**Figure 4:**
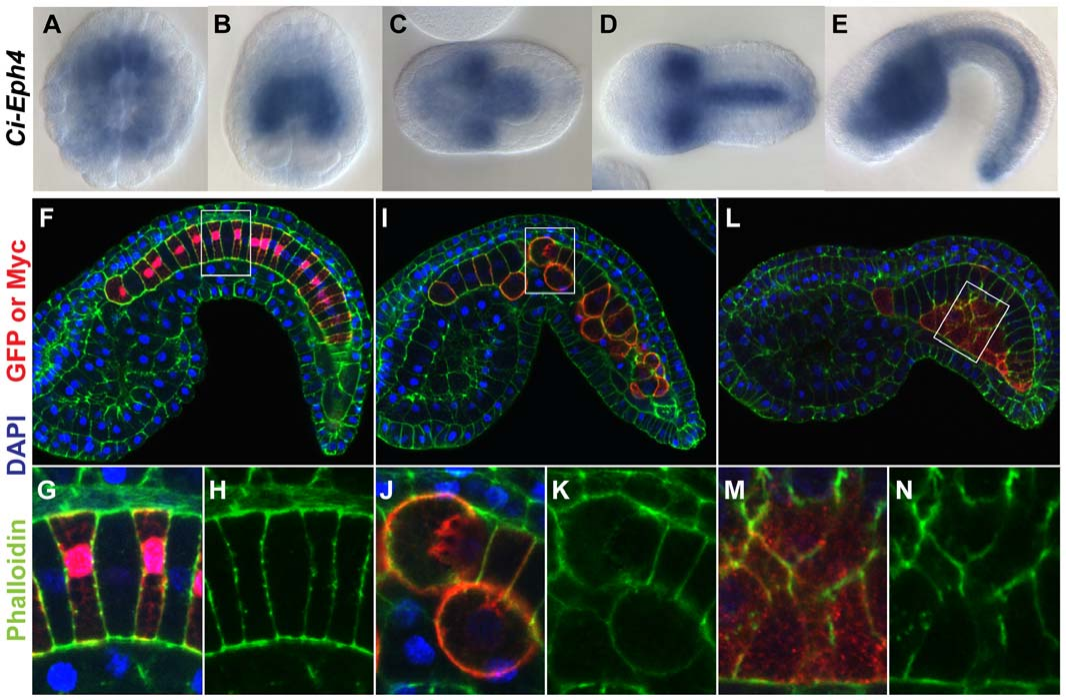
Eph4ΔC misexpression caused morphological defects in notochord cells. (A–E) *In situ* hybridization of early gastrula (A), late gastrula (B), middle neurula (C), early tailbud (D) and middle-late tailbud (E) stage embryos for *Ci-Eph4* demonstrating its preferential expression in notochord lineage cells before and during the cell intercalation process. A is a vegetal view with anterior to the top. B–D are dorsal views with either anterior to the top (B) or to the left (C, D). E is a lateral view with anterior to the left. (F–N) Confocal section images of embryos misexpressing EGFP (F), Eph4ΔC (I) or XDshD2 (L) triple-stained with anti-myc for Eph4ΔC or XDshD2 (red in F and I), phalloidin for actin (red in F, green in F and I) and DAPI for nuclei (blue). Anterior is to the left. (G,J,M) Higher magnification images of the boxed area in F, I and J, respectively. (H,K,N) as G,J,M but only phalloiding signal demonstrating that Eph4ΔC and XDshD2 give rise to defects in morphology and intercalation patterns of notochord cells but phenotypes induced by each transgene are distinct.

Eph/ephrin signaling has been implicated in notochord morphogenesis in zebrafish [12], [13], though it is unknown which step of notochord development is regulated by this signaling. To test whether the same signaling plays a role in dorsoventral patterning of the ascidian notochord, expression profiles of *Eph/ephrin* gene family members in the notochord was examined during convergence and extension. According to ANISEED database (http://139.124.8.91/), only *Ci-Eph3* and *Ci-Eph4* are expressed in notochord lineage cells among *ciona Eph/eprin* gene family members. This is consistent with our whole mount in situ hybridization results (data not shown). Although ANISEED database shows expression of *Ci-ephrinAa* at only early cleavaging stage, we determined that it is not expressed in notochord lineage cells afterward too (Figure S3). It should be noted that expression of *Ci-Eph4* starts to be detected before neurula stage when cell intercalation initiates (Figure 4).

### Dominant-negative forms of Ci-Eph4, but not Dishevelled, disrupt the dorsoventral polarity of the notochord during cell intercalation

**Figure 5:**
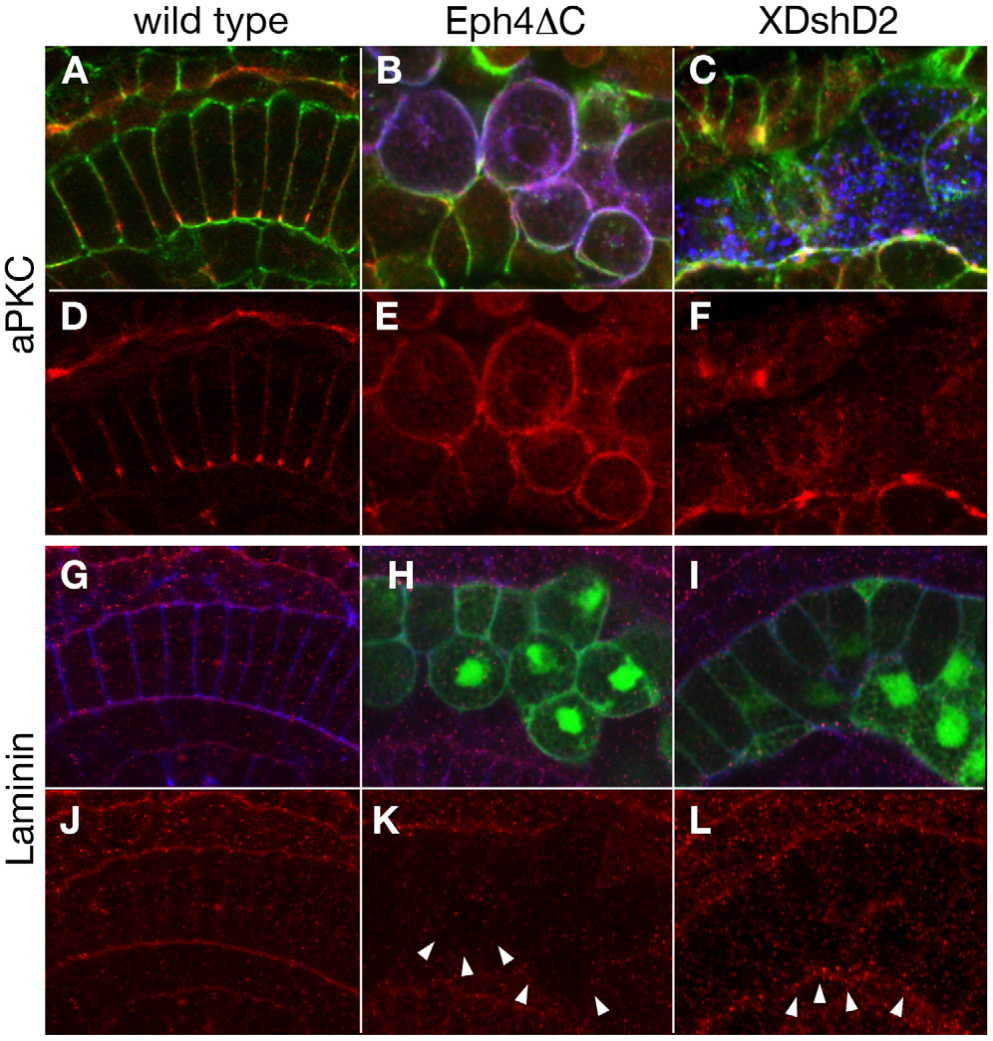
Misexpression of Eph4ΔC but XDshD2 altered dorsovasal polarity of notochord cells. Confocal sagittal section images of middle-late tailbud stage embryos with wild-type (A,D,G,J), Eph4ΔC-expressing (B,E,H,K), and XDshD2-expressing (C,F,I,L) notochord cells, triple-stained with myc-antibody for Eph4ΔC or XDshD2 (blue in B and C), antibodies for aPKC (red in A–F) or Laminin (red in G–L) and phalloidin for actin (green in A–C, blue in G–I). All images are lareral view with anterior to the left. Cells expressing XDshD2 and Eph4ΔC were also detected by co-misexpressed EGFP signal (green in H and I, respectively). Note that Eph4ΔC-expressing notochord cells are segregated from wild type cells and display aPKC on the entire cell surface with no detectable polarity (B,E) and less evident laminin accumulation (arrowheads in K). XDshD2-expressing notochord cells are also segregated from wild type cells but preserve a ventral accumulation of aPKC (C,F) and perinotochordal accumulation of laminin. (arrowheads in L).

**Figure 6:**
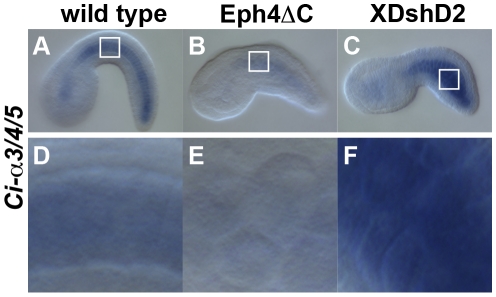
*Ci-lamα3/4/5* transcription was downregulated by misexpression of Eph4ΔC but not by that of XDshD2. *Ci-lamα3/4/5* transcripts detected by in situ hybridizaion in a wild type (A), transfected with Eph4ΔC (B), and transfected with XDshD2 (C) embryos. D, E and F are higher magnification the boxed area in A, B and C, respectively.

To test the possibility that the Eph/ephrin pathway is involved in establishing and/or maintaining the dorsoventral polarity of notochord plate and notochord cells during intercalation, a dominant negative form of Ci-Eph4, Eph4ΔC, which lacks the majority of the intracellular domain, was introduced in notochord cells using a notochord specific promoter element via electropration (Figure 4I–K). The notochord plate and notochord of resulting embryos exhibited a mosaic transgene expression after gastrula stage. In control embryos with only *EGFP* misexpression (n = 126 embryos), all notochord cells, both EGFP positive and negative, exhibited typical coin-shapes and were aligned linearly along the embryonic midline, indicating normal cell intercalation (n = 126 embryos; Figure 4F–H). In contrast, notochord cells of embryos transfected with Eph4ΔC (n = 106 embryos) failed to undergo normal cell intercalation. These cells were rounded and often invaded the surrounding tissue, resulting in an irregular boundary between the notochord and surrounding tissues (n = 106 embryos; Figure 4I–K). Furthermore, Eph4ΔC-misexpressing cells lost asymmetric accumulation of aPKC and instead displayed it over the whole cell surface (Figure 5B,E). The misexpressing cells ectopically located in the surrounding tissues showed diminished levels of both laminin protein (Figure 5H,K) and transcripts (Figure 6B,E). Thus, introduction of Eph4ΔC to notochord cells gave rise to impaired cell polarity and intercalation.

To further control the specificity of induced phenotypes in Eph4ΔC expressing cells, two other mutant forms of Eph, Eph4TM and Eph3ΔC, were used. The former encodes only the transmembrane domain of Ci-Eph4, while the latter encodes a dominant-negative form of Ci-Eph3, which is expressed in notochord cells only after notochord elongation. These mutant proteins were successfully localized at the cell membrane of transfected notochord cells. However, distinct from effects of Eph4ΔC, little or no morphogenetic change was detected in notochord cells with either Eph4TM*-*misexpression (n = 104 embyos) or Eph3ΔC-misexpression (n = 72 embryos) (Figure S4).

Studies in ascidians and other chordates have shown that the planar cell polarity pathway of Wnt signaling also plays a role in notochord morphogenesis [18], [27]. Consistent with these studies, notochord cells transfected with a dominant negative form of Xenopus Dishevelled, XDshD2, showed an incomplete cell intercalation instead forming a packed cell cluster with a smooth border (Figure 4L–N). The XDshD2*-*induced phenotypes were distinct from those seen in Eph4ΔC*-*expressing cells with rounded morphology (Figure 4I–K). Indeed, XDshD2-transfected notochord cells exhibited a ventral accumulation of aPKC (Figure 5C,F) and perinotochordal accumulation of laminin (Figure 5I,L). Furthermore, expression level of *Ci-lamα3/4/5* transcripts was not altered in XDshD2-transfected cells (Figure 6C,F). Thus, a dominant-negative form of Ci-Eph4, but not Dishevelled, disrupts the dorsoventral polarity of notochord cells during cell intercalation although both interfere with normal cell intercalation.

### A dominant-negative form of Ci-Eph4 alters the cleavage angle of notochord cells during the last cell division of notochord cells

**Figure 7:**
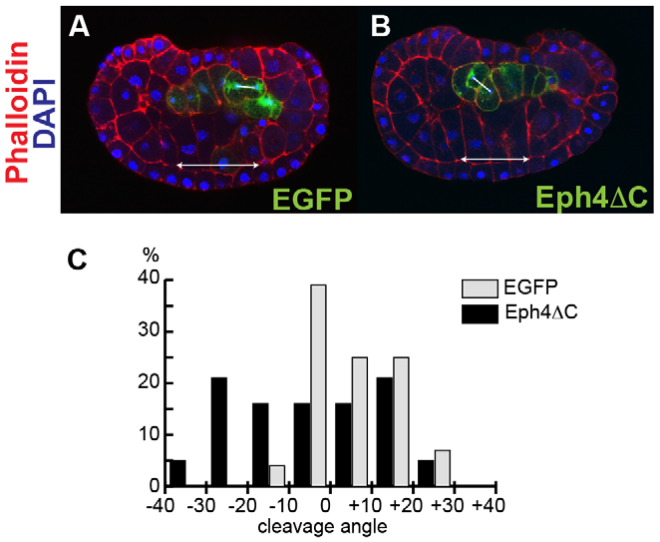
Eph4ΔC alters the cleavage angle of notochord cells. (A–B) The cleavage angle in cells expressing EGFP (A) or Eph4ΔC (B). Cells expressing transgenes were visualized with EGFP signal (green in A) or staining for myc-tag connected to Eph4ΔC (green in B). Embryos were stained for phalloidin (red) and DAPI (blue). Confocal sagittal section images were shown. Anterior is to the left. The angle between a line connecting two centrosomes (lines) in a dividing cell and a line along the basal surface of ventral epidermis (arrows) was measured. (C) Distribution of the cleavage angle in cells expressing EGFP or Eph4ΔC. Samples were grouped based on their cleavage angle and % of each group was shown in a graph.

The above data show that misexpression of Eph4ΔC disrupted the dorsoventral polarity of notochord cells and resulted in abnormal positioning of cells in tailbud stage embryos. Since *Ci-Eph4* became detectable at 110-cell stage when embryos have 10 notochord precursors (which later undergo two additional cell divisions to generate the final 40 notochord plate cells), Eph4ΔC may have affected these early steps of notochord morphogenesis as well. To test this possibility, effects of Eph4ΔC on notochord precursors during their last cell division at the middle neurula stage was examined. Inspection of nuclear morphology and cell shapes identified little or no change in the number of notochord precursor cells in control and Eph4ΔC-expressing embryos (data not shown). Cells expressing Eph4ΔC did not show any notable abnormality prior to the final cell division, and were integrated within the flat and single-layered notochord plate. In their final cell division, however, there was striking difference in the cleavage angle between control and Eph4ΔC expressing notochord cells (Figure 7). The majority of control EGFP-positive cells (n = 28 embryos) divided perpendicular to the notochord plate plane (Figure 7A). In contrast, cells expressing Eph4ΔC (n = 19 embryos) lost the oriented cleavage pattern and exhibited much more random cleavage angles (Figure 7B). The data suggest that Ci-Eph4 signaling begins to function in the notochord cell lineage as early as their last cell division.

## Discussion

In the present study, we show that ascidian notochord cells exhibit asymmetric distribution of apicobasal cell polarity markers along the dorsoventral axis during convergence and extension processes. Importantly, this dorsoventral distribution of cell polarity markers in notochord plate cells is diminished by a dominant-negative form of Eph, but not by that of Dishevelled, giving rise to non-polarized notochord cells that fail to undergo normal intercalation. The data are consistent with a model in which notochord cells establish and maintain a polarity along the dorsoventral axis in an *Eph*-dependent manner restricting cell intercalation along the lateral-to-medial axis.

The notochord of ascidian *Ciona intestinalis* is established though a convergence and extension mechanism mediated by the mediolateral intercalation of postmitotic notochord plate cells [24], [25], [26]. In contrast to well-documented mechanisms that regulate the anteroposterior polarity of the notochord, it remains unclear how notochord cells remain in the notochord plate without delaminating from it along the DV axis. Our finding of an asymmetric accumulation of apicobasal polarity markers, laminin dorsally and aPKC ventrally throughout intercalation suggest that intercalating notochord plate cells are polarized along the dorsoventral axis as seen in the epithelium. However, unlike typical polarized epithelium, localization of these markers changes dynamically in the notochord plate. While aPKC signal becomes restricted to small areas at the ventral midline, laminin signal expands ventrally to surround the notochord. Although spatial resolution of our analysis does not allow us to conclude that production of laminin protein occurs only in aPKC-negative cell surface, our data are consistent with a model presented by Munro and Odell [25], where basolateral cell surface expands ventrally as the notochord plate invaginates. It remains unclear whether the dorsoventral polarity of notochord plate cells is the same or distinct from the apicobasal polarity of the epithelium. However, it is known that laminins and collagen IV are the two most abundant proteins in the basement membrane and play a key role in both apicobasal cell polarity and movement [42], [43], [44], [45], [46], [47], [48]. It is also known that aPKC forms an aPKC/Par3/Par6 ternary complex, and plays a critical role in establishing apical identities of epithelial cells [36]. It would be plausible that these apicobasal-polarity factors play a role in directing intercalation of polarized notochord cells perpendicular to their cell polarity throughout convergence and extension. Consistent with this idea, once intercalation is complete, the dorsoventral polarity of notochord cells disappears: both laminins and collagen IV cover over the entire surface of the notochord (Figure 1, Figure S2 and Figure S5), and aPKC accumulates only at the center of the boundary between neighboring notochord cells.

Our analysis of z-stack reconstruction for aPKC demonstrated that most, if not all, notochord cells showed strong aPKC signal in their ventralmost region. Our data do not support a direct link between the polarity marker localization and boundaries with surrounding tissue types. Each notochord cell has its own neighbors that change overtime with dynamic morphogenetic movement. aPKC signal is detectable initially on the ventral cell surface facing the archenteron, but the signal is still detectable even after the archenteron is replaced by the endoderm. Also, laminin is initially detectable dorsally, but the signal subsequently extends to the boundary between the notochord and more ventral structures such as the muscle and the endoderm. Furthermore, our in situ hybridization data show that *Cs-lama3/4/5* transcripts are highly enriched in the notochord but not in the nerve cord, suggesting that dorsal localization of laminin is not a consequence of a local production of this protein from the nerve cord. Taken together our data support the model in which a developing notochord has an intrinsic cell polarity along the DV axis, which is maintained independently of the neighboring tissue types. How this polarity is established is an important question that should be addressed experimentally in future. It is conceivable that tissue interactions at early developmental stages play a role.

It is currently unknown how asymmetric accumulation of cell polarity marker proteins is regulated in the notochord plate along the dorsoventral axis. Our data suggest that Eph signaling may play a role in this process. The Eph/ephrin signaling pathway is involved in a variety of developmental processes including cell migration, axon guidance and tissue boundary formation in vertebrates [49], [50], [51], [52], [53], [54], [55], [56]. It has also been shown that Eph/ephrin signaling mediates notochord and neural cell fate decision in *Ciona intestinalis* [20]. However, little is known about the role of this signaling in the dorsoventral polarity of notochord plate cells or in their intercalation. Interaction of Eph family receptor protein tyrosine kinases with their membrane-anchored ephrin family ligands induces bi-directional signaling through cell-cell contacts. Binding of ephrin ligands to Eph receptors initiates Eph signaling through Eph dimerization and Eph autophosphorylation on tyrosine residues within the intercellular domain and activation of receptor tyrosine kinase activity. This activation of receptor tyrosine kinase domain causes phosphorylation of tyrosine residues of the partner Eph and downstream target proteins [53], [57]. A truncated form of Eph, which lacks most of the cytoplasmic domain, has been used to inhibit transduction of Eph signaling [21], [22], [58]. Although we showed that misexpression of Eph4ΔC causes abnormal morphogenesis in *Ciona* notochord cells, it remains unclear how endogenous Ci-Eph4 functions in notochord morphogenesis. Five ephrin genes, *Ci-ephrinAa, Ci-ephrinAb, Ci-ephrinAc, Ci-ephrinAd* and *Ci-ephrinB* are annotated in the *Ciona* genome, but no *ephrin* is expressed in notochord cells during convergent extension process (Figure S3 and data not shown, see the ANISEED database, http://139.124.8.91/). Importantly, however, we have found that three types of *ephrin* are expressed in tissues surrounding the intercalating notochord: *Ci-ephrinB* in the endodermal strand during the tailbud stage and *Ci-ephrinAb* and *Ci-ephrinAc* in the neural plate at the late neurula stage (Figure S3 and data not shown, see the ANISEED database, http://139.124.8.91/). Since it has been suggested that cells in the developing nerve chord play a role in directing notochord intercalation [29], it would be conceivable that the regulation of the dorsoventral polarity of notochord plate cells is mediated by interactions of Ci-Eph4 expressed by the notochord with ephrins displayed by these surrounding tissues.

Eph4ΔC-induced notochord phenotypes appear to be distinct from those seen in mutations of planar cell polarity signaling components. In *ciona* notochord cells, components of the planar cell polarity pathway show asymmetric localization along the mediolateral axis during cell intercalation and anteroposterior axis after cell intercalation [27]. Loss of or reduced function of PCP pathway components results in failure to complete cell intercalation but does not affect notochord cell identity [18], [27]. Our data show that a dominant negative form of Dishevelled induces defected intercalation of notochord plate cells but importantly these mutant cells preserve dorsoventral polarity. The data suggest that the planar cell polarity signaling cascade, or at least Dishevelled signaling, regulate notochord morphogenesis mainly along the anteroposterior axis, while the Eph/epherin signaling axis plays a more prominent role in establishing and/or maintaining the polarity along the dorsoventral axis. Our data are consistent with a cell intercalation defect in mutants of another PCP pathway component prickle that have smooth notochord surface [27]. Interestingly, however, these embryos have large patches of laminin staining deep within the notochord [34], which was not detected in notochord cells misexpressing dominant negative dischevelled. It is currently unknow whether this difference is due to a functional difference between these two genes or to difference in approaches for loss-of-function. Further study will be required to explore how individual signaling cascades pattern the notochord along these distinct embryonic axes.

It remains to be determined how reduced Eph activity results in abnormal notochord morphogenesis. Although disturbed cleavage angles in notochord cells expressing Eph4ΔC may not fully explain its drastic effects on cellular positioning and morphology, this data is consistent with the idea that diminished apicobasal polarity results in mispositioning of daughter cells during cell division and affects subsequent cellular intercalation. It should be noted that morphological defects in notochord cells caused by Eph4ΔC resemble those seen in *Cs-lamα/3/4/5* mutants [34]. In both cases, notochord cells exhibit round shape, and fail to undergo normal intercalation. These results suggest that Ci-Eph4 and laminin signaling may interplay in notochord plate cells. Indeed, it has been shown that both laminins and Ephs are downstream of hypoxia-inducible factor-1 transcription factor in mammalian cells [59], [60], suggesting possible transcriptional regulation of laminins mediated by Eph signaling. Consistent with this idea, our data show that both expression level of *laminin* transcripts and accumulation of laminin proteins were reduced in notochord cells expressing Eph4ΔC. Further studies will be necessary to determine whether *laminin* genes are transcriptionally regulated by the Eph/ephrin signaling axis.

An obvious next question would be if the dorsoventral polarity of notochord cells plays a role in notochord morphogenesis of other chordates. Asymmetricity of notochord cells along the embryonic dorsoventral axis has been reported in newt, *Cinops pyrrhongaster*, in which the single cell diameter notochord in *Cinops pyrrhongaster* embryos arises from a single-layered cell sheet by convergent extension movement [61] as seen in *ciona*. In the newt, two antigens whose molecular identity is currently unknown, exhibit localization pattern along the dorsoventral axis [62] resembling that of Ci-lamα3/4/5 [34]. Although it is unknown if these antibodies recognize basement membrane components, this localization pattern suggests that extracellular matrix first forms at the dorsal side of the notochord plate prior to or at the initiation of intercalation. It is plausible that localization of basal characteristics at the dorsal side of the notochord is evolutionarily conserved among organisms whose single cell diameter notochord arises from a single-layered sheet of cell. Further study will be necessary to examine whether the dorsoventral polarity exists in notochord precursor cells and plays an evolutionarily conserved role in notochord morphogenesis of other chordates.

## Materials and Methods

### Embryo Rearing

Adult *Ciona intestinalis* were purchased from Marine Research and Educational Products (M-REP, CA). The animals were kept at 18°C in recirculating artificial seawater. *In vitro* fertilization, dechorionation, and culture of embryos were carried out as described previously [63].

### Cloning of *Ciona intestinalis Eph*, ephrin and *lamα3/4/5* Genes

*Ciona intestinalis Eph, ephrin* and *lamα3/4/5* cDNAs were obtained by RT-PCR from total RNA isolated from early tailbud stage embryos. *C. intestinalis* EST database was used to design the following primer sets: *Ci-Eph1*, 5′-GACCGGAGATTGGAGATCTG-3′ and 5′-GGTGGCATCTCGAACTTGTG-3′; *Ci-Eph2*, 5′-CAAAGAGCCGTCAGCGAATTC-3′ and 5′-CAAATGAAACAATTTAACGGCAG-3′; *Ci-Eph3*, 5′-GCACTCGTTGTGTCAGTTGTTCTAC-3′ and 5′-AACAGTAATCTTTATTAAATAATAAATACAC-3′; *Ci-Eph4*, 5′-TATAATACGAGCGAGAATTTTACAATG-3′ and 5′-CATGCCAGGAAAGTTTAGTACGATTG 3′; *Ci-Eph-like*, 5′-CGTGTTTCTGAAACTCTATAAATG-3′ and 5′-ATCGAGGTGGGTGAGCATAC-3′; *Ci-ephrinAa*, 5′-ACATGTCTCAGCCTGGTTAAG-3′ and 5′-ATTGTATTGTCCGGGTGGTAC-3′; *Ci-ephrinAb*, 5′-CTCTTTGATGGGGTACGAAGCAAG-3′ and 5′-TACTGATCAGCACTTACGTCTATAAC-3′; *Ci-ephrinAc*, 5′-GCACCCGGGATTGCAATTATTATTAG-3′ and 5′-AACAAGTTGTGGCGTGGCATGTTG-3′; *Ci-ephrinAd*, 5′-GAATGGCAACTCAATTTACCTACTAC-3′ and 5′-CACAATACAGTGTGTCGTGGCAAAC-3′; *Ci-ephrinB*, 5′-GCTCGTACCACTTGCGAGTTAAAC-3′ and 5′-ATTCAACATATAGTTTTAAATGGCTATTC-3′; and *Ci-lamα3/4/5*, 5′-AGCACACAAAACCAGTTACAAAGATC-3′ and 5′-AGAGGAGCGGGCTGAGCAGATC-3′. PCR-amplified cDNA fragments were subcloned into pCRII-TOPO vector (Invitrogen, CA) and used as templates to synthesize Digoxigenin-labeled antisense riboprobes for whole-mount *in situ* hybridization.

### Whole-Mount *In Situ* Hybridization

Whole-mount *in situ* hybridization was performed according to [64] with minor modifications; embryos were hybridized at 50°C for 15–18 hr with variable amounts of each probe, ranging from 0.1 to 0.5 g/ml.

### Electroporation

Electroporation was performed according to previously published protocols [63], [65] with some modifications. Fertilized eggs in 100 µl culture solution were mixed with 200 µl of 0.96 M mannitol and 50 µl of 0.25–0.5 µg/µl plasmid DNA solution, transferred to a cuvette with a 4 mm electrode gap and electroporated using the pulse generator ECM 830 (BTX, CA), according to a square pulse protocol (50 V and 16 ms per pulse). Electroporated embryos were allowed to develop further and fixed at various developmental stages in acetone, methanol or 2–4% paraformaldehyde in phosphate-buffered saline (PBS) depending on subsequent analyses.

### Expression Vectors

To generate a notochord-specific expression vector, the *Ci-multidom* gene in the *Ci-Bra*>*Ci-multidom* vector [66] was replaced with a synthetic double-stranded linker fragment encoding PstI, BlpI, SalI, BamHI and NotI sites as follows: 5′-CTGCAGGTATGCTCAGCTCAGTCGACAGAGGATCCGATGCGGCCGC-3′. Another synthetic double-stranded linker fragment which encodes Myc tag as follows 5′-GGATCCGAACAAAAGCTAATTTCTGAAGAAGATCTCGCGGCCGC-3′, was inserted into the resulting vector using BamHI and NotI to generate a construct designated as Bra>PsBlSlBaMycNt. All expression constructs described below were generated by inserting a full or partial coding sequence of *Eph* or *Dishevelled* genes with five glycine linker into Bra>PsBlSlBaMycNt immediately upstream of the Myc-tag sequence. Bra>Eph4ΔN, Bra>Eph4TM and Bra>Eph3ΔN were generated by inserting PCR-amplified cDNAs using SalI and BamHI. The following primer sets were used for PCR-amplification: Bra>Eph4ΔN, 5′-TTGAGTCGACATGGAACAGAAGTATACGAAGTG-3′ and 5′-GTACGGATCCTCCACCTCCGCCTCCAGCGTCAATCTCCTTTGTAAAG-3′; Bra>Eph4TM, 5′-CAGGGTCGACTCACCACTTGAATCTACTACGT-3′ and 5′-GTACGGATCCTCCACCTCCGCCTCCGCAGAACACGATAAGCACGAT-3′; Bra>Eph3ΔN, 5′-TTGAATCGACATGTTTTTTCTATTTATCGTCTTCTACTG-3′ and 5′-GTACGGATCCTCCACCTCCGCCTCCTTGATCAATCTCGGTTGCGATT-3′. The Bra>XDshD2 was obtained by PCR amplification of Ci-Bra-XDsh-D2 [18] with the primer sets 5′-AGAGGCTCAGCATGGCGGAGACTAAAGTGATTT-3′ and 5′-TGTAGTCGACTCCACCTCCGCCTCCCATTGGATTGTAGGAGAGAG-3′ and inserted into the BlpI and SalI sites of Bra>Eph4ΔN. Eph4ΔC-Myc sequence was then replaced with a synthetic double-stranded linker fragment encoding Myc only, using SalI and NotI as follows: 5′- GTCGACGAACAAAAGCTAATTTCTGAAGAAGATCTCGCGGCCGC-3′.

### Immunostaining and Confocal Microscopy

For immunostaining with anti-aPKC antibody, embryos were fixed with 50% and 70% acetone for 3 minutes each at 4°C and 100% acetone for 10 minutes at −20°C. After rehydration through a reverse acetone series, the embryos were washed in phosphate-buffered saline containing 0.1% Tween20 (PBST), blocked with 0.25% blocking reagent (Roche) in PBS, and incubated with the primary anti-aPKC antibody (1∶200) in 0.25% blocking reagent in PBS at 4°C overnight followed with secondary antibodies conjugated with Alexa Fluor 594 goat anti-rabbit IgG (Invitrogen, CA). Immunostaining of laminin with Anti-Cs-lamα3/4/5 [36] or anti-laminin (GeneTex, Inc. GTX11575) antibodies was performed by fixing embryos with 2% paraformaldehyde in PBS for 30 minutes at room temperature, followed by washing in PBST and permeabilization with 20 µg/ml Proteinase K (Ambion) in PBT (0.2% TritonX 100 in PBS) for 5 minutes at room temperature with continuous rocking. After blocking with 5% heat-inactivated goat serum in PBS, embryos were incubated with anti-Cs-lamα3/4/5 antibody (1∶200) or anti-laminin antibody (1∶10) at 4°C overnight. The same protocol was used for staining with anti-c-Myc antibody (Roche) (1∶200), with the following modifications; permeabilization in PBT for 10 minutes, and 0.25% blocking reagent in PBS. Secondary goat anti-mouse antibodies conjugated with either Alexa Fluor 488, 594 or 647 (Invitrogen, CA) antibody was used. Co-immunostaining of aPKC or Cs-lamα3/4/5 with c-Myc was performed using the same fixation and permeabilization methods as described for anti-aPKC and anti-Cs-lamα3/4/5. The nucleus and cell boundaries were visualized with DAPI (Invitrogen, CA) (1∶1000) and phalloidin (Invitrogen, CA) (1∶200) respectively.

### Western blot analysis

Embryos at the early tailbud stage were collected in an Ependorf tube and concentrated by removing supernatant. The embryos were homogenized in 2xsample buffer [0.1 M Tris-HCl (pH 6.8), 4% SDS, 10.2% mercaptoethanol, 20% glycerol, 100 µg/ml Bromophenol blue). Aliquots of proteins equivalent to 2 µl of concelntrated embryo were resolved in 10% SDS-PAGE gel and transferred to Immobilon-PSQ PVDF Membrane (Millipore). Kaleidoscope Prestained Standards (Bio-Rad) was used as a size marker. Anti-Cs-lamα3/4/5 antibody (1∶1000) and anti-laminin (Genetex. Inc.; 1∶100) were used as primary antibodies. The secondary antibody was goat anti-rabbit IgG (H+L) horshradish peroxidase-conjugated (Amersham; 1∶10000). Signals were detected using Chemi-Lumi One L (Nacalai) and the ImageQuant LAS4000 imager (GE Healthcare).

## Supporting Information

Figure S1 Localization pattern of laminin over the notochord surface detected by using anti-mouse laminin antibody. Embryos at late neurula (A, D), early tailbud (B,E) or middle-late tailbud (C,F) stages double-stained with the anti-mouse laminin (D–F) and with phalloidin (A–C). Images were taken under a convensional optical microscope (A,D) or a confocal microscope (B,C,E,F). White dashes encircle notochord forming region. Accumulation of Laminin at the dorsal side of the notochord is indicated by blank arrowheads in D. Lateral view, anterior is to the left. (G) Western blot analysis with ant-Cs-lam (left) and mouse laminin (right) antibodies. Cs-laminin antibody showed a strong signal over 200 KDa (arrowhead) whereas mouse laminin antibody showed a weak signal at a similar size (open arrowhead) but also showed many other bands.
(4.98 MB TIF)

Figure S2 Subcellular distribution of aPKC in notochord cells after cell intercalation process. Confocal sagittal section images of an embryo immunostained for aPKC (red) and stained with phalloidin (green) at the early larva stage (A) or late larva stage (D). A reconstructed cross section image at the level indicated by arrows in A and D is shown in B/C and E/F, respectively.
(2.21 MB TIF)

Figure S3 Expression of *ephrinAa* detected by *in situ* hybridization. Late neurula (A) and middle-late tailbud (B) stage embryos. Lateral view, anterior is to the left.
(1.19 MB TIF)

Figure S4 Misexpression of Eph4TM or Eph3ΔC causes no severe morphological defects in notochord cells. Confocal section images of embryos misexpressed with Eph4TM (A) or Eph3ΔC. Embryos were stained with phalloidin (green) and DAPI (blue). Cells expressing myc-tagged Eph4TM or Eph3ΔC were visualized by immunostaining for myc (red). Lareral view. Anterior is to the left. Dorsal is to the top.
(1.27 MB TIF)

Figure S5 Localization pattern of collagen IV over the notochord surface. An embryo at late middle-late tailbud stages stained for the anti-mouse collagen IV (B) and with phalloidin (A). Images were taken under a confocal microscope. Lateral view, anterior is to the left.
(1.17 MB TIF)

Movie S1 A Z-stack of confocal sagittal section images of a late-neurula stage embryo shown in Figure 3A stained with anti-aPKC antibody (red) and phalloidin (green). Signal of aPKC in the ventral side of the notochord is indicated by arrows.
(6.09 MB MOV)

Movie S2 A Z-stack of confocal sagittal section images of an early-tailbud stage embryo shown in Figure 3D stained with anti-aPKC antibody (red) and phalloidin (green). Signal of aPKC in the ventral side of the notochord is indicated by arrows.
(7.06 MB MOV)
