# Supplementary figures and images for: Eph Regulates Dorsoventral Asymmetry of the Notochord Plate and Convergent Extension-Mediated Notochord Formation
Source: PLoS One. 2010 Oct 29;5(10):e13689. doi: 10.1371/journal.pone.0013689 (PMC2966392; doi:10.1371/journal.pone.0013689)

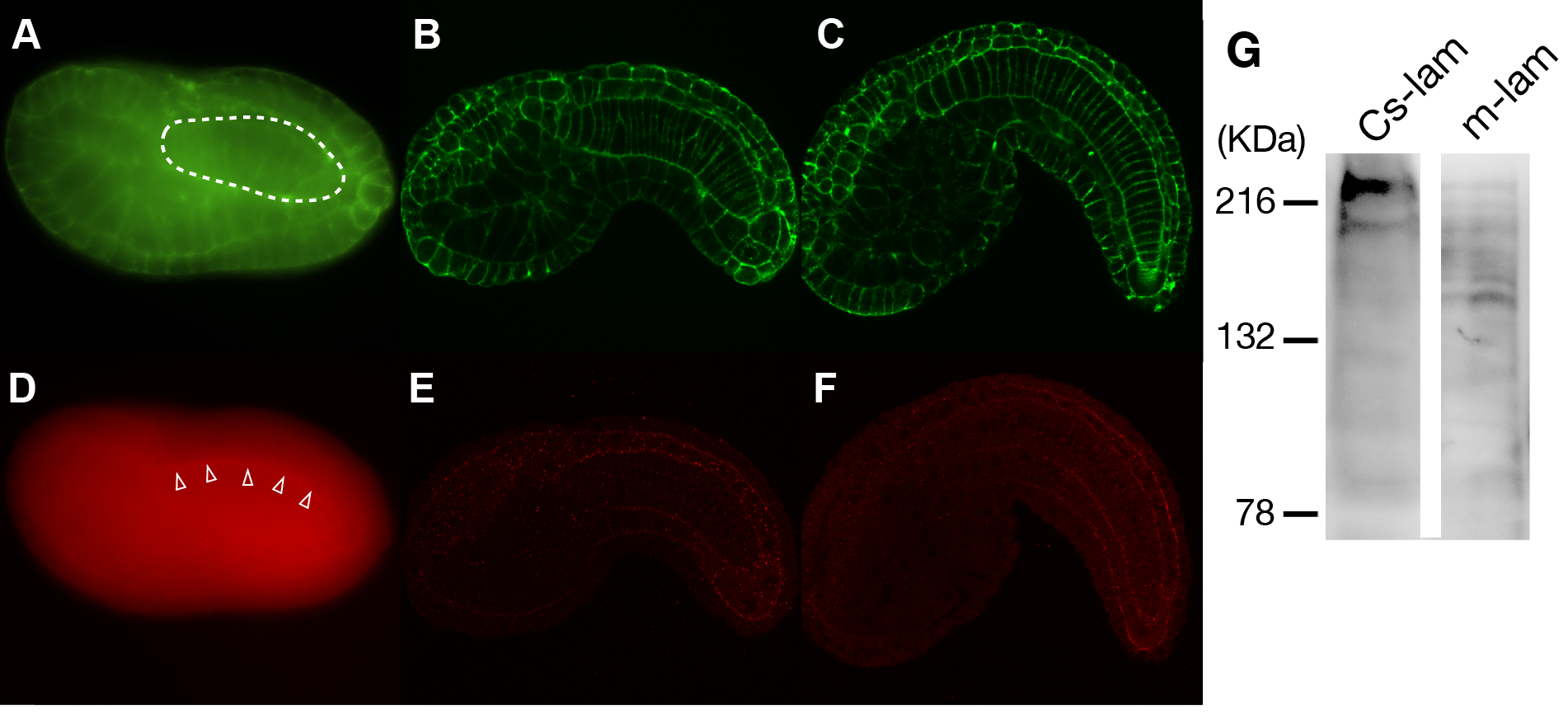

Supplement: Figure S1 — Localization pattern of laminin over the notochord surface detected by using anti-mouse laminin antibody. Embryos at late neurula (A, D), early tailbud (B,E) or middle-late tailbud (C,F) stages double-stained with the anti-mouse laminin (D–F) and with phalloidin (A–C). Images were taken under a convensional optical microscope (A,D) or a confocal microscope (B,C,E,F). White dashes encircle notochord forming region. Accumulation of Laminin at the dorsal side of the notochord is indicated by blank arrowheads in D. Lateral view, anterior is to the left. (G) Western blot analysis with ant-Cs-lam (left) and mouse laminin (right) antibodies. Cs-laminin antibody showed a strong signal over 200 KDa (arrowhead) whereas mouse laminin antibody showed a weak signal at a similar size (open arrowhead) but also showed many other bands. (4.98 MB TIF) [file pone.0013689.s001.tif]

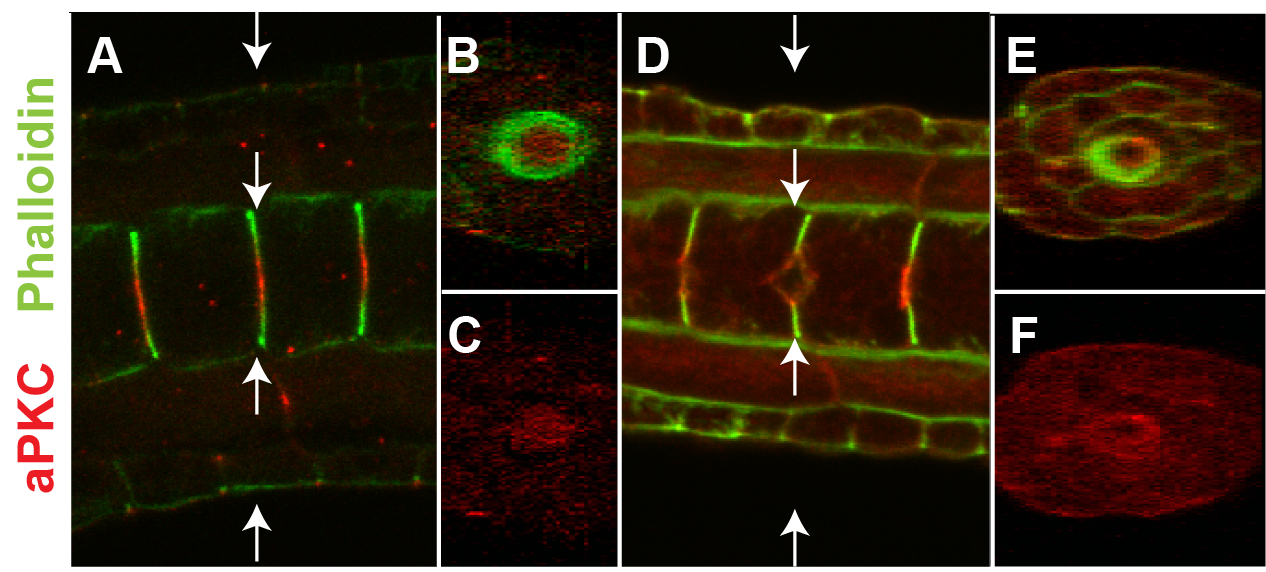

Supplement: Figure S2 — Subcellular distribution of aPKC in notochord cells after cell intercalation process. Confocal sagittal section images of an embryo immunostained for aPKC (red) and stained with phalloidin (green) at the early larva stage (A) or late larva stage (D). A reconstructed cross section image at the level indicated by arrows in A and D is shown in B/C and E/F, respectively. (2.21 MB TIF) [file pone.0013689.s002.tif]

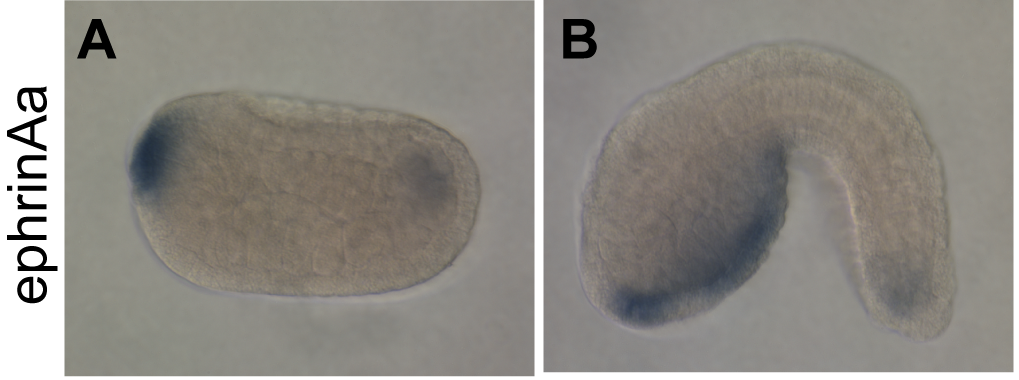

Supplement: Figure S3 — Expression of ephrinAa detected by in situ hybridization. Late neurula (A) and middle-late tailbud (B) stage embryos. Lateral view, anterior is to the left. (1.19 MB TIF) [file pone.0013689.s003.tif]

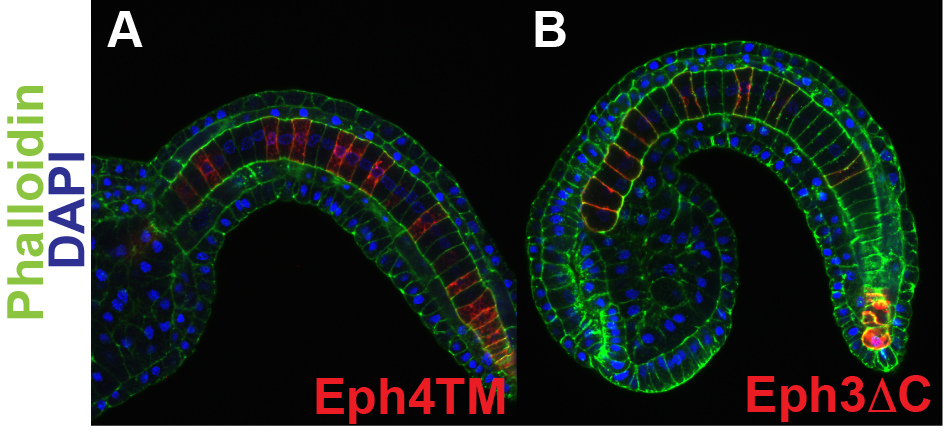

Supplement: Figure S4 — Misexpression of Eph4TM or Eph3ΔC causes no severe morphological defects in notochord cells. Confocal section images of embryos misexpressed with Eph4TM (A) or Eph3ΔC. Embryos were stained with phalloidin (green) and DAPI (blue). Cells expressing myc-tagged Eph4TM or Eph3ΔC were visualized by immunostaining for myc (red). Lareral view. Anterior is to the left. Dorsal is to the top. (1.27 MB TIF) [file pone.0013689.s004.tif]

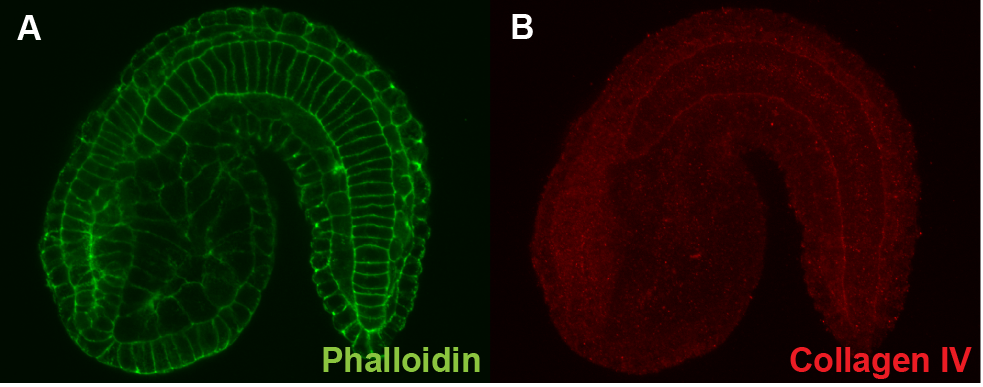

Supplement: Figure S5 — Localization pattern of collagen IV over the notochord surface. An embryo at late middle-late tailbud stages stained for the anti-mouse collagen IV (B) and with phalloidin (A). Images were taken under a confocal microscope. Lateral view, anterior is to the left. (1.17 MB TIF) [file pone.0013689.s005.tif]
